# A brief interactive training with medical students improves their diabetes knowledge about hypoglycemia

**DOI:** 10.1186/s12909-019-1615-x

**Published:** 2019-05-28

**Authors:** Elizabeth A. Beverly, Marilyn D. Ritholz, Rochelle G. Rennie, Sophia C. Mort

**Affiliations:** 1 Department of Family Medicine, Ohio University Heritage College of Osteopathic Medicine, Athens, OH 45701 USA; 2 The Diabetes Institute, Ohio University, Athens, OH 45701 USA; 3 Joslin Diabetes Center, Boston, MA USA; 4 Harvard Medical School, Boston, MA USA; 5 Department of Medicine, Ohio University Heritage College of Osteopathic Medicine, Athens, OH 45701 USA; 6 The Graduate College, Translational Biomedical Sciences Program, Ohio University, Athens, OH 45701 USA

**Keywords:** Diabetes, Hypoglycemia, Glucagon, Medical education

## Abstract

**Background:**

Hypoglycemia is a severe clinical problem with physical and psychosocial implications for people with type 1 and type 2 diabetes. Medical students would benefit from formal education on how to treat hypoglycemia as well as how to administer glucagon in case of a severe hypoglycemic emergency. The purpose of this study was to assess the effectiveness of a brief training to improve medical students’ knowledge and attitudes about diabetes, hypoglycemia, and glucagon administration.

**Methods:**

We conducted a feasibility study to assess the effectiveness of an interactive training session on diabetes education with an emphasis on hypoglycemia. We measured medical students’ knowledge and attitudes toward diabetes, hypoglycemia, and glucagon before and after the training. We performed Chi-Square tests, paired t-tests, determined effect sizes using Cohen’s *d*, and analyzed short answer responses via content and thematic analyses.

**Results:**

Two hundred and seventeen participants (age = 25.1 ± 2.3 years, 45.2% female, 78.3% white, 36.4% planned to pursue primary care, response rate of 94.3%) completed surveys. Following the training, participants’ total knowledge scores improved by five percentage points to 82.6 ± 11.0% (t-value = 7.119, *p* < 0.001). We also observed positive improvements in the General Test scores to 82.3 ± 12.6% (t-value = 5.844, *p* < 0.001) and Insulin Use Test scores to 82.4 ± 17.4% (t-value = 4.103, *p* < 0.001). For the hypoglycemia test, participants averaged 55.7 ± 24.8% pre-training and 83.0 ± 22.4% post-training (t-value = 14.258, p < 0.001). Lastly, participants scored 87.6 ± 18.5% on the glucagon test after the training session. In addition, we observed positive improvements in all five diabetes attitudes subscales after the training, with the largest magnitude of change in the “Psychosocial impact of diabetes” subscale (t-value = 9.249, *p* < 0.001, Cohen’s *d* = 0.60). Qualitatively, more participants recognized the severity of hypoglycemia after the training. They also learned how to approach diabetes from the patient’s perspective and valued the clinically relevant and practical information provided during the training session, such as the “15–15 Rule.”

**Conclusions:**

Medical students need to learn about patients’ everyday experiences of diabetes in order to have an understanding of and confidence to assess and treat hypoglycemia. These findings underscore the importance of training medical students on how to actively assess and manage the risk of hypoglycemia in people with diabetes.

## Background

Diabetes is a devastating disease affecting more than 30 million Americans [1] and 425 million people worldwide [2]. Because of its chronic and evolving nature, the condition is debilitating to our health and economy, costing the United States (US) $327 billion in 2017 [3]. This equates to diabetes accounting for 1 out of every 4 health care dollars spent in this country. Hypoglycemia is a significant yet often unrecognized contributor to these healthcare costs. A single episode of hypoglycemia requiring the assistance of a healthcare provider costs an average of $1161 [4], while an episode of hypoglycemia treated by a non-medical third-party costs an estimated $66 and a self-managed episode costs $11 [4]. Individuals with type 1 diabetes (T1D) experience an average of two episodes of hypoglycemia per week and one to two severe hypoglycemic events annually [5]. For people with type 2 diabetes (T2D), the frequency of hypoglycemia varies by treatment, with hypoglycemia occurring most frequently with insulin therapy [6, 7]. However, recent research by Gehlaut [8] and colleagues shows that hypoglycemia may be more common than previously thought in people with T2D, with 49.1% of participants having one hypoglycemic episode in a five day period, and of those, 75.4% experiencing hypoglycemia unawareness [8]. This is of particular concern because most persons with T2D are treated by non-specialists, such as primary care providers who may not fully understand the risks associated with hypoglycemia [9]. These statistics underscore the importance of finding innovative ways to detect, treat, and prevent hypoglycemia through education and research.

Hypoglycemia is the leading adverse effect of intensive diabetes management for people with diabetes [10–13]. A missed meal, too much exercise, alcohol, or not enough food for the amount of insulin administered can lead to hypoglycemia [14]. If not treated immediately, hypoglycemia can become severe. Level 1 hypoglycemia is defined as a blood glucose level < 70 mg/dL (3.9 mmol/L) and level 2 is < 54 mg/dL (3.0 mmol/L) [15]. Commons signs and symptoms of hypoglycemia include shakiness, nervousness, sweating, blurred vision, confusion, fatigue/sleepiness [16]. Treatment of hypoglycemia requires the ingestion of glucose-containing foods, preferably pure glucose [15]. The American Diabetes Association recommends the “15–15 Rule” or consuming 15 g of carbohydrate to raise one’s blood glucose and checking it after 15 min [16]. If a person’s blood glucose level remains < 70 mg/dL, they should repeat the “15–15 Rule” [16]. However, in cases of severe hypoglycemia (level 3) hypoglycemia, defined by symptoms of loss of consciousness, seizure, coma, or death, a person may require assistance due to altered mental and/or physical state [15]. For example, a person may be physically unable to eat or drink a rapid acting source of glucose or they may be unconscious. In this circumstance, a person will need a glucagon injection to restore blood glucose levels to normal [15]. Fortunately, glucagon emergency kits are easily available, and those in close contact with a person prone to hypoglycemia should be instructed on how to administer glucagon.

Medical students are a subpopulation who would benefit from formal education on how to treat hypoglycemia as well as how to administer glucagon in case of a severe hypoglycemic emergency. In the same way that medical students complete requisite basic life support training during their undergraduate medical education, they could participate in a brief training that focuses on hypoglycemia treatment and glucagon administration. A one-time training that provides an overview of diabetes self-management education and support, the definitions of hypoglycemia levels 1–3, the “15–15 Rule,” and glucagon demonstration may be an approach to achieve this goal. Thus, the purpose of this study was to assess the effectiveness of a brief training to improve medical students’ knowledge and attitudes about diabetes, hypoglycemia, and glucagon administration. We hypothesized that the training would increase diabetes knowledge, in particular knowledge of hypoglycemia and glucagon, and improve attitudes toward diabetes.

## Methods

This feasibility study evaluated the effectiveness of an interactive lecture in an Endocrine and Metabolism course with medical students. Specifically, we measured second year medical students’ knowledge and attitudes toward diabetes before and after an interactive training in order to 1) assess changes in knowledge pre- and post-training, 2) assess changes in attitudes pre- and post-lecture, and 3) explore perceived severity toward hypoglycemia. The Ohio University Office of Research Compliance approved the protocol (Institutional Review Board #19-E-1) and all recruitment procedures and materials.

### Participants

Second year medical students enrolled at a large medical school with three campuses were invited to participate in an online, anonymous assessment before and after an interactive training on diabetes education, with an emphasis on hypoglycemia. Students completed the pre- and post-assessment prior to receiving any material on diabetes so that they would provide a baseline viewpoint of their diabetes knowledge and attitudes. The research team distributed the assessment via email on January 4, 2019; a reminder email with the assessment was distributed 3 days later. The post-assessment was distributed immediately following the training and completed in the lecture rooms on January 9, 2019. Participation in the study was voluntary.

### Training on hypoglycemia treatment and glucagon administration

The training was developed as an alternative to a standard didactic lecture that included the definition of hypoglycemia, a list of signs and symptoms of hypoglycemia, and a list of food and drinks to correct for hypoglycemia presented via PowerPoint presentation; estimated time dedicated to the topic was 10 min. The two-hour interactive training covered a brief overview of the material delivered in diabetes self-management education and support: the disease process, healthy eating, regular physical activity, blood glucose monitoring, medication management, psychosocial factors, and hypoglycemia treatment. An experienced behavioral diabetes researcher trained in interactive lecturing delivered the training. The participants were encouraged to interact with each other and the lecturer. The lecturer incorporated straightforward and rhetorical questions to engage the audience as well as a group training exercise. Audiovisual techniques, including a PowerPoint presentation, a hypoglycemia treatment kit (i.e., glucose tablets, glucose gel, juice boxes, cheese crackers, blood glucose meter, alcohol wipes, tissues, bandages, rubber gloves), and demonstration of an emergency glucagon administration training kit followed by group practice with the kit, were used to train medical students on the treatment of hypoglycemia. Glucagon administration kits were available on all three campuses.

### Measures

In addition to sociodemographic factors (age, sex, race/ethnicity, rural/urban locale where participant grew up) and planned specialty choice, participants completed the following measures:

*Revised Diabetes Knowledge Test (DKT2)* [17] is a 23-item test that assesses level of knowledge for adults with T1D and T2D. Participants were instructed to complete this measure as if they had diabetes. The DKT2 consists of two parts. The first part is a general knowledge part (GKP) and contains 14 questions; the second part is the insulin use part (IUP) and contains nine questions. Both parts were included in this study for a global DKT (GDKT). The DKT2 demonstrates acceptable reliability for the GKP (α = 0.77) and good reliability for the IUP (α = 0.84) [17]. An additional 9 knowledge questions were created for the purposes of this study to assess knowledge about hypoglycemia (4 questions) and glucagon administration (5 questions). The four hypoglycemia questions were included in the pre-assessment; however, the five glucagon questions were not included. The research team made this decision to prevent participants from searching for answers about the glucagon questions after the pre-survey in order to accurately assess their knowledge immediately after the training session.

*Diabetes Attitude Scale-3 (DAS-3)* [18], a 33-item scale that measures diabetes-related attitudes with five discrete subscales: 1) “Need for special training”, 2) “Seriousness of type 2 diabetes”, 3) “Value of tight glucose control”, 4) “Psychosocial impact of diabetes”, and 5) “Attitude toward patient autonomy”. Participants are asked to rate their level of agreement on a 5-point Likert scale, ranging from strongly agree = 5 to strongly disagree = 1. The scale demonstrates superior subscale reliability scores and high content validity [18].

Participants also completed a series of short answer questions in the pre-survey including, 1) “Have you had any personal experiences with diabetes or exposure to diabetes among your family and friends? Please explain.” and 2) “In your own words, how severe is diabetes and hypoglycemia?” Short answer questions in the post-survey included the following,1) “In your own words, how severe is diabetes and hypoglycemia?” and 2) “What did you learn from the training? What was helpful? What was not helpful?”

### Data collection

Participants completed the anonymous survey online via the electronic survey service Qualtrics (Provo, UT: Qualtrics). Qualtrics permitted the research team to download students’ survey responses into a spreadsheet without including identifying information (i.e. email address, name). To link participants’ pre- and post-survey responses, we included three questions at the beginning of the survey which served as a unique identifier (i.e., favorite ice cream flavor, favorite animal, the number of the day in the month they were born); this unique identifier has been successfully used by the research team in previous studies to protect participant anonymity. All participants provided informed consent via the online survey prior to participation. No researchers were present when potential participants decided to participate or decline in order to ameliorate any perceived pressure to have to participate. Students with questions about the study were directed to email or phone the principal investigator (EAB). Participation in the survey lasted approximately 10–15 min.

### Data analysis

We assessed demographic factors using descriptive statistics and presented them as means and standard deviations or sample size and percentages. We performed paired t-tests to examine changes in DKT2 and DAS-3 before and after the interactive lecture to assess changes in diabetes knowledge and attitudes. In addition, we determined effect sizes using Cohen’s *d* by calculating the mean difference between the pre- and post-survey responses divided by the pooled standard deviation. We defined statistical significance as a *p*-value less than 0.05, and conducted analyses in SPSS statistical software version 25.0 (Chicago, IL: SPSS Inc.).

We analyzed the open-ended short answer questions via content and thematic analyses [19]. First, two researchers (EAB, RGR) independently marked and categorized key words, phrases, and texts to identify codes that described participants’ experiences with diabetes and their perceived severity of diabetes and hypoglycemia. The researchers revised, discussed, and resolved coding discrepancies through consensus to establish inter-coder reliability [20]. The Cohen’s kappa coefficient for the interrater agreement between the two coders was 0.959, indicating almost perfect agreement. [21, 22] A total of 215 (99.1%) participants completed the pre-training open-ended question about the severity of diabetes and hypoglycemia and 209 participants (96.3%) completed the post-training open-ended question. However, 21 participants did not comment on hypoglycemia on the pre-training question and 11 did not comment on the post-training question, and therefore were not included in the analysis. Considering the high number of responses, we conducted Chi-Square analyses to determine if participants’ perceived severity of hypoglycemia changed before and after the training.

Second, we conducted thematic analysis to identify patterns across the data [23, 24]. The selected themes described participants’ understanding of hypoglycemia as well as their experiences with the interactive training. We derived themes from data that occurred multiple times, both within and across short answer responses. Each theme includes words expressed by the participants to demonstrate that the concepts were grounded in the data.

## Results

Of the 230 students invited to participate in the study, 217 completed the survey for a response rate of 94.3%. The mean age of the participants was 25.1 ± 2.3 years, 45.2% (*n* = 98) identified as female, 78.3% (*n* = 170) identified as white, 43.3% (*n* = 94) grew up in a town (i.e., 2500–50,000 people), and 36.4% (*n* = 79) planned to pursue primary care (see Table 1). Of the 217 students, 135 (62.2%) reported personal experiences with individuals diagnosed with diabetes. Twenty-six participants (12.0%) reported having a first-degree relative diagnosed with T1D or T2D (i.e., parent, sibling), 42.4% (*n* = 92) had a second-degree relative (i.e., aunt, uncle, cousin, grandparent), and 30.0% (*n* = 65) had a friend.

**Table 1:** Participants’ Demographic Characteristics (*n* = 217)

Variable	All participants n (%)
Age (years)	25.1 ± 2.3
Sex	
Female	98 (45.2)
Male	119 (54.8)
Hispanic/Latino	5 (2.3)
Race	
Asian	18 (8.3)
Black/African American	14 (6.5)
Mixed	8 (3.7)
Other	7 (3.2)
White/Caucasian	170 (78.3)
Community grew up in:	
Major Metropolitan Area (Over a million people)	15 (6.9)
Metropolitan Area (500,001 to 1,000,000 people)	13 (6.0)
City (100,001 to 500,000 people)	43 (19.8)
Small City (50,001 to 100,000 people)	32 (14.7)
Town (2500 to 50,000 people)	94 (43.3)
Rural Area (fewer than 2500 people)	20 (9.2)
Medical school campus	
Athens	124 (57.1)
Cleveland	48 (22.1)
Dublin	45 (20.7)
Plan to pursue primary care	
Yes	79 (36.4)
No	138 (63.6)

Mean scores to the DKT2 are presented in Table 2. Prior to receiving any content or training on diabetes, the participants scored an average of 77.5 ± 13.2% on the knowledge test. On the DKT2 subscales, participants scored a 77.6 ± 13.3% on the General Test and a 77.3 ± 18.9% on the Insulin Use Test. Following the training, participants’ total knowledge scores improved by five percentage points to 82.6 ± 11.0% (mean improvement = 5.05, t-value = 7.119, *p* < 0.001; see Table 2). We also observed positive improvements in the General Test scores to 82.3 ± 12.6% (mean improvement = 4.73, t-value = 5.844, *p* < 0.001) and Insulin Use Test scores to 82.4 ± 17.4% (mean improvement = 5.13, t-value = 4.103, p < 0.001). For the four–question hypoglycemia test, participants averaged 55.7 ± 24.8% pre-training and 83.0 ± 22.4% post-training (mean improvement = 27.26, t-value = 14.258, p < 0.001; see Tables 2 and 3). The largest magnitude of change occurred with the hypoglycemia test pre- and post-training, with a Cohen’s d of 1.16, indicating a very large effect. With this effect, participants scored 1.16 standard deviations higher on their post-training hypoglycemia test score compared to their pre-training test score. Lastly, participants scored 87.6 ± 18.5% on the five-question glucagon test after the training session; this was the highest average score out of all of the knowledge tests (see Tables 2 and 3).

**Table 2:** Mean Differences between Diabetes Knowledge Test-2 Scores Pre- and Post-Training (*n* = 216)

	Pre-Panel (%)	Post-Panel (%)	*p*-value	Cohen’s *d*
Total Score	77.5 ± 13.2	82.6 ± 11.0	< 0.001	0.42
General Test	77.6 ± 13.3	82.3 ± 12.6	< 0.001	0.36
Insulin Use Test	77.3 ± 18.9	82.4 ± 17.4	< 0.001	0.28
Hypoglycemia Test (*n* = 210)	55.7 ± 24.8	83.0 ± 22.4	< 0.001	1.16
Glucagon Test (n = 210)	–	87.6 ± 18.5	–	–

**Table 3:** Hypoglycemia and Glucagon Test Questions Pre- and Post-Training (n = 210)

	Pre-training n (% correct)	Post-training n (% correct)	*p*-value
Hypoglycemia Test Questions			
1. Which of the following is a symptom of mild/moderate hypoglycemia?	121 (55.8)	143 (67.8)	< 0.001*
a. alertness			
**b. blurry vision**			
c. loss of appetite			
d. flat affect			
2. Which of the following is a sign of severe hypoglycemia?	153 (70.5)	188 (89.1)	< 0.001*
a. unusual bleeding			
b. sudden intense headache			
c. edema			
**d. seizures**			
3. How many grams of carbohydrate should be given to treat mild/moderate hypoglycemia?	129 (59.4)	180 (85.3)	0.003*
a. 5 g			
**b. 15 g**			
c. 20 g			
d. 30 g			
4. In case of a hypoglycemic emergency, what should be administered by a healthcare professional or trained volunteer?	81 (37.3)	189 (89.6)	0.113^ǂ^
a. rapid-acting insulin			
b. long-acting insulin			
c. glucose			
**d. glucagon**			
Glucagon Test Questions			
5. Glucagon administration kits come prepared as:	–	187 (88.6)	–
a. pre-mixed solution			
**b. vial of glucagon dry powder and separate sterile liquid in a syringe kit**			
c. both types of kits			
6. When withdrawing the prescribed amount of glucagon back into the syringe, the solution must be:	–	196 (92.9)	–
a. white			
**b. clear**			
c. colloidal solution			
7. Clean injection sites include all of the following EXCEPT:	–	170 (86.3)	–
a. thigh			
b. upper arm			
**c. abdomen**			
d. buttock			
8. A common symptom a person may experience after a glucagon administration is:	–	196 (97.0)	–
**a. headache**			
b. shortness of breath			
c. abdominal pain			
d. numbness or tingling of extremities			
9. Once the person with diabetes is awake and able to drink, they should be given:	–	177 (83.9)	–
a. water or diet soda			
**b. regular soda or juice**			
c. no food or drink			

Note. Values were missing for post-training follow-up (n = 7); *p-values derived from Chi-Square tests; ^ǂ^*p*-value derived from Fisher’s Exact Test ^ǂ^

Responses to the five DAS-3 subscales are presented in Table 4. Pre-training mean scores showed participants generally agreed with the “Need for special training” (4.53), “Seriousness of type 2 diabetes” (4.07), “Value of tight glucose control” (3.97), “Psychosocial impact of diabetes” (4.28), and “Attitude toward patient autonomy” (4.13; see Table 3). Participants with personal experiences with diabetes reported more positive attitudes for “Seriousness of type 2 diabetes” (mean difference = 0.14, t-value = 2.250, *p* = 0.025), “Value of tight glucose control” (mean difference = 0.15, t-value = 2.298, *p* = 0.023), and “Psychosocial impact of diabetes” (mean difference = 0.14, t-value = 2.396, *p* = 0.017) compared to participants without personal experiences.

**Table 4:** Mean Differences between Diabetes Attitude Scale Subscale Scores Pre- and Post-Training (*n* = 212)

	Pre-Panel	Post-Panel	*p*-value	Cohen’s *d*
Need for special training	4.53 ± 0.41	4.65 ± 0.41	< 0.001	0.32
Seriousness of type 2 diabetes	4.07 ± 0.46	4.19 ± 0.49	< 0.001	0.25
Value of tight glucose control	3.97 ± 0.47	4.07 ± 0.49	0.001	0.21
Psychosocial impact of diabetes	4.28 ± 0.43	4.54 ± 0.43	< 0.001	0.60
Attitude toward patient autonomy	4.13 ± 0.40	4.30 ± 0.43	< 0.001	0.41

Following the training, we observed positive improvements in diabetes attitudes for all five subscales (see Table 4): “Need for special training” (mean improvement = 0.12, t-value = 4.166, *p* < 0.001, *n* = 212); “Seriousness of type 2 diabetes” (mean improvement = 0.12, t-value = 3.647, p < 0.001); “Value of tight glucose control” (mean improvement = 0.11, t-value = 3.373, *p* = 0.001); “Psychosocial impact of diabetes” (mean improvement = 0.27, t-value = 9.249, p < 0.001); and “Attitude toward patient autonomy” (mean improvement = 0.17, t-value = 6.261, p < 0.001). We observed the largest magnitude of change with the “Psychosocial impact of diabetes” subscale, with a Cohen’s d of 0.60 indicating a medium effect (see Table 3).

With the pre-training open-ended question, 78.9% (*n* = 153) rated hypoglycemia as “very severe”, 18.0% (*n* = 35) rated hypoglycemia as “relatively severe”, and 3.1% (*n* = 6) rated it as “not severe” Following the training session, 88.1% (*n* = 192) viewed hypoglycemia as “very severe”, 2.3% (*n* = 5) found it to be “relatively severe”, and one participant (0.5%) viewed it as “not severe”. A Chi-Square test revealed that the training was associated with a change in participants’ perception of hypoglycemia severity pre- and post-training assessment, with more participants rating it as “very severe” (*Χ*^2^ = 49.700; *p* < 0.001). Further, in participants who rated hypoglycemia as “not severe” or “relatively severe” pre-training, we observed positive improvements in diabetes attitudes post-training in “Need for special training” (mean improvement = 0.15, t-value = 2.000, *p* = 0.047, *n* = 194) and “Seriousness of type 2 diabetes” (mean improvement = 0.21, t-value = 2.703, *p* = 0.007, n = 194).

### Qualitative themes

#### Recognizing the severity of hypoglycemia

Following the training session, the majority of participants (88.1%; *n* = 192) emphasized the serious and life-threatening consequences of hypoglycemia. Many referred to the severe signs and symptoms of level 3 hypoglycemia, or that the person with diabetes requires assistance due to altered mental and/or physical state [15], as evidenced in the following quotations:

“Diabetes is extremely severe as it affects every aspect of a patient’s life. Hypoglycemia is even more severe as a patient can go into seizures or coma.” (ID 46)

“Both are super severe. Diabetes affects all aspects of life. Hypoglycemia can lead to seizures and death. Crazy scary stuff!” (ID 76)

“They are very severe conditions that could have severe consequences and even lead to death if not treated properly” (ID 108).

Participants noted not only the serious medical symptoms, but also the psychosocial symptoms of trauma. For example, they were aware that loss of consciousness and being close to death could be viewed as a traumatic event: “Hypoglycemia is also traumatizing due to potential for coma and death” (ID 65). They recognized that this traumatic experience could lead to fear of hypoglycemia.

Lastly, participants valued the content delivered in the training. Many participants appreciated that the topic of hypoglycemia, and its three levels, was covered in the training session because it is not addressed adequately in the medical school curriculum. Considering the high prevalence of diabetes in the US and worldwide, participants thought more healthcare providers should learn and discuss hypoglycemia:

“Diabetes and hypoglycemia can be very severe. It is often not talked about as much as it should be among health care providers or in our case medical students. But it can be the difference between life and death.” (ID 214)

“Diabetes is a disease which can range in severity from mild to extreme, depending on many factors. It is important for diabetes patients to be aware of their condition and what factors improve or worsen their condition and do what they can to control them. Hypoglycemia itself can also range from mild to life-threateningly severe, People with diabetes and people who know and live with them should be trained to respond to hypoglycemic episodes.” (ID 40)

#### Learning clinically relevant and practical information

Of the 217 participants, 209 (96.3%) provided short answer responses to the question about what they learned from this training session. The vast majority (*n* = 205, 98.1%) reported positive experiences with the training. Most stated that they learned practical information, such as how to treat hypoglycemia and administer glucagon. They agreed that the “15–15 Rule” and hands-on practice with the glucagon emergency kit was beneficial:

“The Glucagon kit information was totally new as well as the 15-15 rule being a useful reminder tool.” (ID 1)

“Learning how to inject glucagon was very important and something I did not know beforehand.” (ID 15)

“I learned about the treatment for hypoglycemia. 15-15 was a new concept for me. The actual administration of the glucagon was also helpful.” (ID 131)

Participants also learned how to look at diabetes and its management from the perspective of the patient. This training offered participants a glimpse into the daily lives of people with diabetes, and not simply the signs and symptoms for diagnosis and medications for treatment. Participants were taught diabetes self-care behaviors, psychosocial factors entailed in living with diabetes, and treatment for hypoglycemia. This may have facilitated a greater understanding of diabetes and the challenges people with diabetes face, as expressed by these two participants:

“I thought this training was helpful in learning how to think from the perspective of someone with diabetes and the added stress a chronic disease brings to their lives. I think it is very important to acknowledge this because a significant portion of the population is affected by diabetes and healthcare professionals need to know how to educate, manage and treat this disease. It was also helpful to learn clinically relevant things like how to treat hypoglycemia.” (ID 18)

“The main thing I learned is how to manage hypoglycemic emergencies. I also learned how to look at diabetes from the perspective of the patient and understand how hard it can be to manage it.” (ID 12)

Finally, several participants wrote comments supporting the need to understand hypoglycemia treatment. They commented on how offering this training to those who live or work with persons with diabetes was essential and important. In addition, they felt that the severity of hypoglycemia warranted training for all people regardless of whether or not they were in the medical field. Participants also believed that this training was clear and straightforward, and therefore, could be understood by the general population:

“Learning how to use and administer a glucagon kit was very helpful, and something that I think everyone should learn.” (ID 154)

“The glucagon administration training can be given to non-medical personnel that has access to patients that may experience a hypoglycemic emergency!” (ID 119)

## Discussion

In this feasibility study, we assessed second year medical students’ knowledge and attitudes toward diabetes before and after an interactive training on diabetes education with a focus on hypoglycemia. Prior to the training, participants held positive attitudes towards diabetes and an average understanding of diabetes knowledge; however, their knowledge of hypoglycemia was limited. Following the training, participants’ knowledge of diabetes and hypoglycemia increased and participants answered the majority of glucagon questions correctly. In addition, all five diabetes attitudes subscales improved, with the largest effect observed with the “Psychosocial impact of diabetes” subscale. Qualitatively, more participants recognized the severity of hypoglycemia after the training. They also learned how to approach diabetes from the patient’s perspective. Lastly, participants valued the clinically relevant and practical information provided during the training session, including the “15–15 Rule” and how to administer glucagon. These findings support the inclusion of diabetes education with an emphasis on hypoglycemia treatment in medical education.

Participants in our study supported the need for more healthcare providers to learn about and discuss hypoglycemia; this recommendation is supported by other studies of medical students who were exposed to diabetes education. For example, a study assessing medical students’ confidence in treating diabetes emergencies where mortality and morbidity could be high also reported a strong recommendation for further training in all aspects of diabetes care [25]. Another study of an all-day education program that integrated lectures and case-based learning on diabetes acute care for medical students working on inpatient units found that their program increased students’ knowledge for diagnosing and managing hypoglycemia and increased their confidence in treating hypoglycemia [26]. Our participants valued the simple and practical information offered on diabetes management and hypoglycemia as well as how to administer glucagon. Prior research supports interactive teaching when educating students with novel information [27]. Clinical recognition of hypoglycemia and its treatment is typically unfamiliar for medical students; therefore, incorporating interactive, lecture-based teaching methods into the medical school curriculum is recommended [27]. Next steps in our research need to explore how our students are able to utilize the knowledge they have acquired during this brief training in their future clinical work. Interestingly, a recent survey study assessing 1003 US physicians’ experience and knowledge of hypoglycemia for adults with T2D found that hypoglycemia knowledge was highly correlated with correct therapeutic decision-making [28], which strongly suggests that understanding and treating hypoglycemia may play a major role in developing healthcare providers’ clinical skills.

Our participants also reported that they learned how to look at diabetes and its management from the perspective of the patient. One important element of a patient-centered approach includes individualizing treatment, which is important when treating patients with diabetes because fear of hypoglycemia or actual hypoglycemia demands the need to know both how to treat hypoglycemia and individualize treatment to prevent its occurrence [29]. For example, sometimes, a higher Hemoglobin A_1c_ is recommended for those with hypoglycemic unawareness, chronic kidney disease, cardiovascular disease, or older age [30]. In addition, a patient-centered approach also includes shared medical decision-making among provider, patient, and family. Patients with diabetes need to feel as though their medical as well as psychosocial experiences are included in therapeutic decisions, but this may not always occur. For example, in a qualitative study of patients’ experiences of living with hypoglycemia, participants reported that physicians did not inquire about the ways hypoglycemia affected their feelings about themselves, their family relationships, or their work but rather focused solely on the biomedical features of hypoglycemia [31]. This underlines the importance of including psychosocial inquiry for the treatment of hypoglycemia in medical education programs.

Importantly, our brief interactive training, helped the medical students in this study learn about the seriousness of diabetes and the life threatening nature of hypoglycemia as well as the psychosocial impact of these conditions on the person with diabetes as well as on his/her family and friends. Our participants’ qualitative responses revealed that they became aware of how patients’ experience of a near-death event such as severe hypoglycemia may be seen as traumatic and can interfere with the person’s future adherence to treatment. For example, patients may develop a fear of hypoglycemia, which could manifest in omitting or decreasing insulin in an attempt to avoid hypoglycemia [32]. Thus, these findings highlight the importance of teaching medical students about both the physical manifestations of hypoglycemia and the possible psychosocial sequelae, which can affect ongoing treatment adherence.

Participants also reported increased understanding about how to assess and treat hypoglycemia for the patient as well as for his/her family. They noted how the severity of hypoglycemia warranted training for all people regardless of whether or not they were in the medical field. Research studies support the idea of including family members in hypoglycemic management and prevention [29, 33, 34]. One study found that hypoglycemia takes its emotional toll on family members and that living with a person who has T1D and hypoglycemic unawareness contributes to family members’ increased worry, anxiety and their own traumatization [33]. Interestingly, another study showed that high levels of worries about hypoglycemia were not associated with family members’ involvement in diabetes care but were associated with increased odds of the relative attending diabetes-related visits to healthcare providers [34]. Therefore, healthcare providers need to be aware of and address not only the treatment and prevention of hypoglycemia for patients, but also understand the impact on their families. It may be important to encourage family members of patients who experience frequent hypoglycemia attend diabetes-related office visits. Again, a patient-centered approach may improve treatment and prevention of hypoglycemia by allowing for a discussion of the psychosocial factors affecting all parties.

Finally, in this age of technology, it is necessary to acknowledge the world of diabetes technology and its effect on hypoglycemia. Research shows that Continuous Glucose Monitors (CGMs) have revolutionized the prevention and early treatment of hypoglycemia [29] These devices that measure interstitial glucose levels every 5 min and provide real time data, allow for early detection and preemptory treatment of hypoglycemia [29, 35]. However, most physicians have not been trained in the interpretation of CGM data and the use of those data for generating recommendations for diabetes self-management [35] Thus, there is a need for guidelines for physicians, as to when to use these new systems for control of insulin administration [35] Again, this suggests the strong need for continued medical education on diabetes and hypoglycemia for physicians throughout their professional lives.

### Limitations

Limitations of this study include data from one medical school, selection bias, social desirability bias, and lack of a control group. Data from one medical school limits the generalizability of findings to other programs, although this medical school has three campuses with very different geographical regions (rural, suburban, and urban). Further, the Endocrine and Metabolism course is delivered during the second year of medical school, thus limiting our ability to enroll students in the first, third, or fourth years. Next, our findings may be susceptible to selection bias, as students who volunteered to participate may have been more willing or motivated to answer questions about diabetes, hypoglycemia, and glucagon. However, we reported a very high response rate (94.3%), which decreases the risk for selection bias and increases the reliability and validity of our findings. In addition, the responses, particularly the open-ended questions, may be susceptible to selection bias given participants may have felt undue pressure to provide positive feedback on the training session. Finally, this study presents findings from a training session on diabetes education with an emphasis on hypoglycemia. We did not include an attention control condition as a comparison group. Future research should use a randomized-control design to assess the impact of two different educational interventions on medical student knowledge and attitudes towards diabetes and hypoglycemia: a one-time training session versus an attentional control session (e.g., hypertension using the new American College of Cardiology and the American Heart Association guidelines).

## Conclusion

This study highlights the importance of exposing medical students to a patient-centered approach to diabetes care. Medical students need to learn about patients’ everyday experiences of illness, and since diabetes is so prevalent in today’s world they need to have an understanding of and confidence to assess and treat hypoglycemia, an acute and serious complication of diabetes. Although the participants in this study were at the beginning of their medical education, through the use of interactive learning, they were able to learn and benefit from a brief and focused educational training. These findings underscore the importance of training medical students on how to actively and adequately assess and manage the risk of hypoglycemia in people with diabetes [9]. Additional research comparing this training to the standard teaching content on hypoglycemia is needed to determine if the interactive hypoglycemia and glucagon training is more effective in educating students and impacting patient outcomes.

## Abbreviations

DAS-3: Diabetes Attitude Scale-3
DKT2: Revised Diabetes Knowledge Test
T1D: Type 1 diabetes
T2D: Type 2 diabetes
US: United States
